# Applying the scientific method when assessing the influence of migratory birds on the dispersal of H5N1

**DOI:** 10.1186/1743-422X-4-132

**Published:** 2007-12-04

**Authors:** Paul L Flint

**Affiliations:** 1 U.S. Geological Survey, Alaska Science Center, 1011 East Tudor Road, Anchorage, Alaska, 99503, USA

## Abstract

**Background:**

The role of wild birds in the dispersal of highly pathogenic avian influenza virus H5N1 continues to be the subject of considerable debate. However, some researchers functionally examining the same question are applying opposing null hypotheses when examining this issue.

**Discussion:**

I describe the correct method for establishing a null hypothesis under the scientific method. I suggest that the correct null hypothesis is that migratory birds can disperse this virus during migration and encourage researchers to design studies to falsify this null. Finally, I provide several examples where statements made during this debate, while strictly true, are not generally informative or are speculative.

**Summary:**

By adhering to the scientific method, definitive answers regarding the role of wild birds in the dispersal of highly pathogenic viruses will be reached more effectively.

## Background

Considerable debate remains regarding the role of wild birds in the dispersal of the highly pathogenic avian influenza (HPAI) H5N1 virus. Numerous articles have been published on this topic, many of which lack any data which would allow critical testing relevant to this issue. Throughout the literature, there are two opposing views with regard to the assumptions regarding to the role of wild birds in dispersing H5N1. First, some authors assume that wild birds cannot disperse this virus over long distances and cite the lack of studies demonstrating such movements [1-4]. Conversely, other authors presume that wild birds can disperse this virus [5-8]. These two opposing views are based on reversal of the functional null hypothesis. The goal of this paper is to apply the scientific method to the development of the appropriate null hypothesis for this issue.

## Discussion

### The most appropriate null hypothesis

The implied null hypotheses are Ho(1): Birds can disperse H5N1 during migration; and Ho(2): Birds cannot disperse H5N1 during migration. Both are potentially valid null hypotheses and each uses the other as the alternative. Thus the main question becomes, which is more appropriate as a working null hypothesis? The development of a null hypothesis, under the scientific method, is predicated on the desire to control the probability of making a type I error. Recall that type I errors occur when a null hypothesis is rejected when in fact the null hypothesis is true [9]. Thus, choosing between these two potential null hypotheses requires assessing which of the alternative type I errors is more severe. Type I error from Ho(1): Conclude that migratory birds cannot disperse H5N1 when in fact they do; Type I error from Ho(2): Conclude that migratory birds do disperse H5N1 when in fact they do not. Because there are currently no data available that would allow a critical test of either of these hypotheses we are left with considering which of these type I errors is most severe or unacceptable. Since the primary interest in H5N1 is relative to the potential risk to the poultry industry and human health, it would seem that prematurely dismissing a potential carrier for dispersal has the potential to allow this virus to expand its range undetected as well as hinder the response to detected outbreaks. Conversely, incorrectly concluding that migratory birds can disperse this virus is simply inefficient. That is, time and resources may be wasted sampling wild birds to detect virus dispersal that is not occurring. However, this inefficiency is only relevant to human health risks if associated resources would be re-directed to issues associated with alternative pathways of H5N1 dispersal (i.e., by poultry transport). Given the funding processes associated with these programs, such re-allocation would seem unlikely. Accordingly, Ho(1) is the appropriate working null hypothesis as the type I error based on this null hypothesis is the most severe and the probability of making this error should be minimized and controlled. While acknowledging that under the strict scientific method, a null hypothesis is never accepted as being true (that is, null hypotheses can only be falsified), it is common practice, particularly in the field of wildlife biology where researchers have little control over potential covariates, to functionally assume the working null as being true while actively attempting to falsify the null. Researchers should pursue data which will allow a critical test of the null hypothesis that wild birds can disperse H5N1 during migration; particularly long distance migration. Importantly, sufficient resources need to be allocated to this effort such that failure to reject this null is associated with reasonable statistical power (i.e., minimizing the probability of a type II error where we fail to reject a null that is false)

### Uninformative conclusions drawn from valid results

Finally, throughout the debate on the respective roles of migratory and domestic birds in the dispersal of H5N1, there are numerous statements that are not based on data presented, or when they are based on data, are not necessarily conclusive. Two such examples are detailed below. First, it has been concluded that wild birds are not an important carrier because HPAI viruses such as H5N1 are rarely isolated from apparently healthy wild birds [10]. However, the same statement can be made about domestic chickens in areas where H5N1 is considered endemic [5]. Thus, the same logic, as applied to migratory birds, could be used to conclude that domestic poultry are not an important carrier. In a second example, it has been speculated that wild birds could not effectively transport H5N1 over long distances as migratory performance would be negatively influenced by the infection [4]. While others pointed out that H5N1 appeared to kill wild birds nearly as efficiently as domestic poultry and noted that "dead ducks don't fly" [1]. However, many of the same arguments would likely apply to long distance transport of domestic poultry. During transport, domestic birds are frequently deprived of food and water for extended periods and are exposed to extreme environmental conditions. Again, the same logic could be used to conclude that exposed domestic poultry are less likely to survive long distance transport for the same immunological reasons. In both of these examples, while the original statements may be strictly true, the conclusions drawn from them are not necessarily valid or informative.

## Conclusion

As the debate regarding the role of wild birds and domestic fowl in the dispersal of H5N1 continues, researchers should be careful to draw conclusions directly from data (i.e., avoid speculation) and ensure that conclusions are empirically and logically supported by all available data. By following the scientific principles, collecting data required for critical tests of hypotheses, and avoiding speculation, definitive conclusions regarding the dispersal of H5N1 will be reached more effectively.

## Abbreviations

HPAI: highly pathogenic avian influenza

## Competing interests

The author(s) declare that they have no competing interests.
